# Gait Speed Is Not Associated with Vasogenic Shock or Cardiogenic Shock following Cardiac Surgery, but Is Associated with Increased Hospital Length of Stay

**DOI:** 10.1155/2018/1538587

**Published:** 2018-10-23

**Authors:** Kimmie Clark, Taylor Leathers, Duncan Rotich, Jianghua He, Katy Wirtz, Emmanuel Daon, Brigid C. Flynn

**Affiliations:** ^1^Medical Student, University of Kansas School of Medicine, Kansas, KS, USA; ^2^Graduate Student, Department of Biostatistics, University of Kansas Medical Center, Kansas, KS, USA; ^3^Biostatistician, Department of Biostatistics, University of Kansas Medical Center, Kansas, KS, USA; ^4^Quality Outcomes Coordinator, Department of Nursing, University of Kansas Medical Center, Kansas, KS, USA; ^5^Assistant Professor, Department of Cardiac Surgery, University of Kansas Medical Center, Kansas, KS, USA; ^6^Associate Professor, Department of Anesthesiology, University of Kansas Medical Center, Kansas, KS, USA

## Abstract

**Objective:**

Frailty has been associated with adverse outcomes following cardiac surgery. Gait speed has been validated as a marker of frailty. Slow gait speed has been found to be associated with mortality after cardiac surgery. However, it is unknown why slow gait speed predisposes to cardiac surgical mortality.

**Design:**

A retrospective analysis.

**Participants:**

Patients undergoing cardiac surgery who had a 5-meter walk test performed preoperatively (n=333 of 1735 total surgical patients) from January 2013 to March 2017.

**Setting:**

A tertiary care academic hospital.

**Measurements and main results:**

Gait speeds were stratified by tertiles: <0.83 m/s, 0.83–1 m/s, and >1 m/s. There was no difference in the incidence of cardiogenic or vasogenic shock when comparing the gait speed groups. Total hospital length of stay was significantly different among the gait speed groups (*p*=0.0050). Also, patients in the slowest gait speed tertile had a significant association with need for a postoperative permanent pacemaker (*p*=0.0298).

**Conclusion:**

There was no significant association between gait speed and the incidence of cardiogenic or vasogenic shock after cardiac surgery. Gait speed was associated with increased hospital length of stay and need for a permanent pacemaker after cardiac surgery.

## 1. Introduction

Frailty is a syndrome of increased vulnerability to stressors caused by multiple impairments in organ function and diminishment of physiological reserves [1]. Frailty, measured by various instruments, has been shown to be a prognostic indicator of poor outcomes after cardiac surgery, including perioperative [2–4] and one-year mortality [5]. It has also been shown that gait speed is a validated and reliable marker of frailty [6–8].

Slow gait speed is an accurate prognostic indicator of outcomes after cardiac surgery when compared to other measures of frailty [9]. However the reasons for this have yet to be explored. Understanding *why* gait speed is associated with increased cardiac surgical risk is paramount when attempting to optimize frail patients prior to surgery as scrutiny concerning healthcare quality, costs, and outcomes are of current relevance.

Since shock is a known cause of poor outcomes after cardiac surgery [10], we undertook an exploratory study investigating if gait speed is associated with either postoperative vasogenic or cardiogenic shock. Identifying the mechanisms in which gait speed is associated with adverse outcomes may lead to preventative measures aiming at attenuating these risk factors. To the authors' knowledge, this is the first study investigating the *etiology* of poor outcomes following cardiac surgery in patients in terms of gait speed.

## 2. Methods

Following institutional review board approval, we undertook a retrospective analysis of patients undergoing cardiac surgery in our tertiary care center during January 2013–March 2017. We analyzed the 5-meter walk test which is administered as a routine preoperative assessment during the cardiac surgical preoperative workup for patients who have a clinic visit prior to surgery at our hospital.

Patients were excluded from the study if the 5-meter gait speed walk test was not documented. Common reasons for nondocumentation of gait speed was urgent or emergent surgery, transfer from outside hospital, in-patient admission prior to surgery, other reasons precluding a preoperative clinic visit, inability to walk unassisted, or lack of ancillary staff on the day of the clinic visit. Patients were not included in the study if on preoperative inotropic or mechanical assisted support.

**Figure 1:**
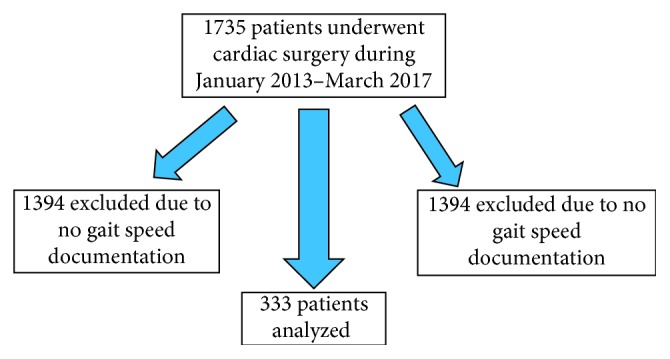
Schematic representation of enrollment process including the two exclusion criteria.

In all, 341 of the 1,735 patients who underwent cardiac surgery during the study period had documented 5-meter gait speeds. Patients undergoing transcatheter aortic valve replacement (TAVR) were excluded due to the fact that none had cardiopulmonary bypass, leaving 333 patients for analysis (Figure 1).

The 5-meter gait speed walk test was administered with the following guidelines:

1. Instruct to “walk at your comfortable pace” until a few steps past the 5-meter mark
2. Begin each trial with the word, “go”
3. Start the timer with the first footfall after the 0-meter line
4. Stop the timer with the first footfall after the 5-meter line
5. Repeat 3 times and record average allowing sufficient time for recuperation between trials.

Vasogenic shock was defined as requirement of a continuous infusion of norepinephrine, vasopressin, and/or phenylephrine in the intensive care unit postcardiac surgery in order to maintain our ICU standard of care mean arterial pressure of at least 65 mmHg. This was maintained per nursing titration order set. Cardiogenic shock was defined as the need for one or more of the inotropic agents used in our ICU for inotropic support: epinephrine, milrinone and/or dobutamine, or the need for an intraaortic balloon pump in order to maintain cardiac index >2 L/minute, or rarely based on echocardiographic findings of need for inotropic support. Patients with both shocks were those requiring inotropic support and vasopressor medication in order to meet the predefined blood pressure and cardiac index goals.

Secondary outcomes included 30-day mortality, incidence of both cardiogenic and vasogenic shock, intensive care unit length of stay, hospital length of stay, reoperations for any cardiac reason, prolonged ventilation (defined as >24 hours after surgery) postoperative atrial fibrillation, postoperative need for a pacemaker, acute kidney injury (AKI) (defined as an increase of ≥50% in creatinine from preoperative creatinine), surgical site infection, and postoperative stroke. Preoperative systolic heart failure is defined as an ejection fraction <55%.

## 3. Statistical Analysis

No sample size calculation was done before designing this study as adequate information was not available for power analysis. Patient characteristics were summarized with descriptive statistics at three frailty levels defined by gait speed (slow, medium, and fast) and compared across the frailty levels. The association of each outcome with frailty was examined using chi-squared or Fisher's exact test (categorical outcomes) or Kruskall–Wallis test (continuous outcomes). The significance level for the two primary outcomes is 0.025 to control the overall type I error rate at 0.05. No adjustment of multiple tests was considered for secondary outcomes, and the test results are considered exploratory.

## 4. Results

Mean gait speed for the entire cohort was 1 m/s (standard deviation (SD) 0.17). For analysis, gait speeds were divided into three tertiles based on previous research [9] and analyzation of the gait speeds in our cohort, which showed similar natural cutoff points of <0.83 m/s, 0.83–1 m/s, and >1 m/s.  Table 1 displays the baseline characteristics of the patients divided by the three gait speed tertiles. Society of Thoracic Surgeons (STS) scores (*p*=0.0019), preoperative congestive heart failure (*p*=0.0410), and preoperative chronic lung disease (*p*=0.0003) were different across the three gait speed groups.

**Figure 2:**
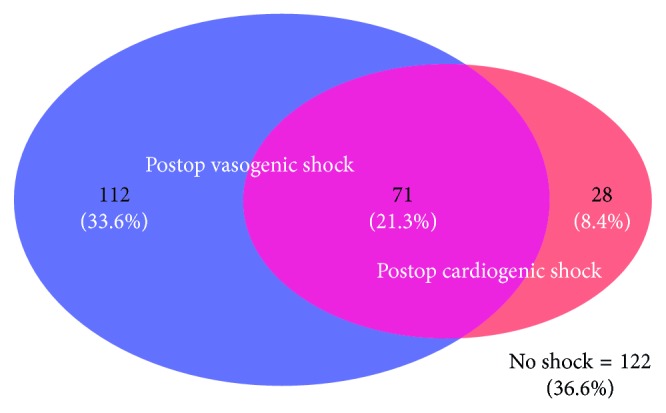
Diagram detailing the number and proportion of 211 patients with postoperative isolated vasogenic (blue), isolated cardiogenic shock (pink) and both shocks (purple). Of the 333 patient cohorts, 122 patients had no postoperative shock.

**Figure 3:**
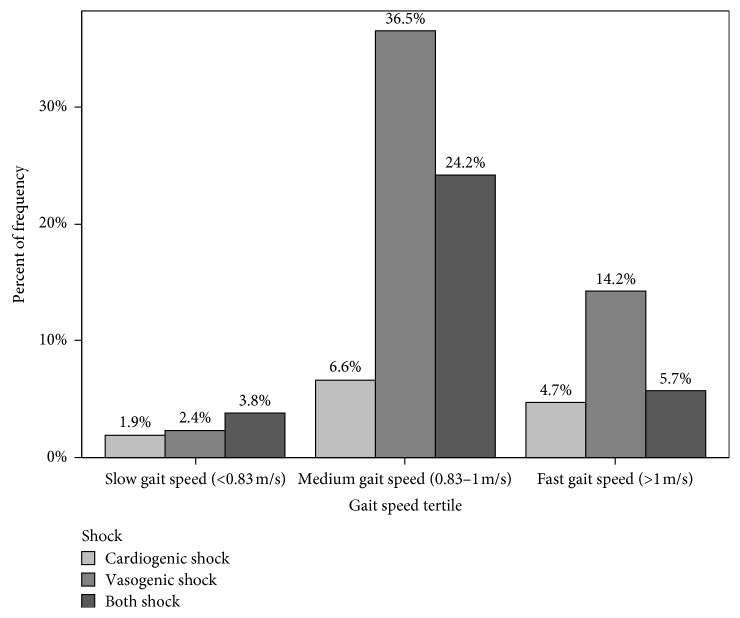
Bar graph illustrating the percentage of patients with each type of shock (isolated vasogenic, isolated cardiogenic, or both shocks) (*n*=211) within each gait speed tertile. Not shown are the 122 patients (40.7%) who experienced no postoperative shock.

Figure 2 illustrates the incidence of postoperative vasogenic shock (*n*=183; 55%), cardiogenic shock (*n*=99; 29.7%), and both shocks (*n*=71; 21.3%).  Figure 3 details the number of patients within each gait speed tertile with vasogenic, cardiogenic, or both shocks. There was no association with gait speed and either vasogenic shock (*p*=0.0805) or cardiogenic shock (*p*=0.3832) (significance level of 0.025 after multiple comparison adjustments) (Table 2). The rate of vasogenic shock was not significantly different in any of the gait speed groups.

Total hospital length of stay was significantly different among the gait speed groups (*p*=0.0050). Also, patients in the slowest gait speed tertile had a significant association with need for a postoperative permanent pacemaker (*p*=0.0298). Length of stay in the ICU and postoperative AKI was not different among the gait speed tertiles; however, AKI was most prominent in the slowest gait speed tertile.

As for other secondary outcomes, gait speed was not associated with mortality. There were proportionately more deaths (6.5% in slowest gait speed tertile versus 1.4% and 2.3% in the medium and fast gait speed tertiles, respectively) in patients with slow gait speeds. Although the difference seems clinically meaningful, it is not statistically significant likely due to the small number of deaths (*n*=7, mortality rate 2.1%). We did find that patients who developed postoperative cardiogenic shock had a significantly higher rate of 30-day mortality (*p*=0.0033) (Table 3).

## 5. Discussion

Frailty conceptually is defined as diminished capability to recover from pathologic or iatrogenic stressors due to aging-related impairments [11, 12]. However, advancing age does not necessary equate to frailty. The pathobiology of frailty involves dysregulation of the immune, endocrine, and metabolic systems [13].

While this dysregulation is part of normal aging, there may be differences in healthy cellular senescence and apoptosis in patients who have significant frailty. Eventually metabolic and nutritional deficiencies followed by muscle mass loss lead to a frail person who can more easily succumb to an acute stress such as surgery [11]. This combined with other common comorbidities may lead to inadequate vascular tone and/or cardiac function in the postoperative period.

To our knowledge, this is the first investigation evaluating the mechanisms that cause gait speed to be an independent predictor of adverse outcomes, including mortality, following cardiac surgery [9]. The current study did not demonstrate a significant association of any gait speed tertile with postoperative vasogenic or cardiogenic shock (0.0805 and 0.3832, respectively). It appears that the development of postoperative shock is multifactorial without an obvious relationship to how fast one ambulates preoperatively. Additionally, we found no differences in intraoperative factors that can predispose to postoperative shock, such as the type of cardiac surgery, CPB time, preoperative ejection fraction, congestive heart failure, peripheral arterial disease, or need for reoperation.

Preoperative gait speed did have a significant association with hospital length of stay (*p* = 0.0050). A recent study found that increased length of stay following cardiac surgery was associated with postoperative gait speed [14], but did not look at the preoperative variable. We believe preoperative gait speed is much more meaningful when considering preoperative risk evaluation and potentially incorporating gait speed as an adjunct in a risk calculating model, such as the Society of Thoracic Surgeons (STS) risk model [15].

Since this is the first study identifying preoperative gait speed as a marker of frailty to be associated with hospital length of stay following cardiac surgery, future investigation is needed. Previous studies have associated frailty identified by frailty scoring systems, not gait speed, to be associated with in-hospital mortality and major morbidity [16]. Additionally and pertinent to our study, frailty has been associated with increased rates of institutional discharge after cardiac surgery [2].

Although this study did find an increased need for pacemaker placement postoperatively, this is unlikely to have affected hospital length of stay. In our institution, pacemakers are readily placed as early as postoperative day one if the surgeon anticipates conduction abnormalities resulting from the surgical procedure. If the patient displays a sinus pause or significant conduction abnormality, a pacemaker is placed expeditiously thus not to delay discharge.

It is this transition of frail patients out of the hospital that likely takes more planning than in patients who are not frail. Even to return home with frequent family visits or part-time in-home health assistance requires logistical and financial planning that needs to be done prior to hospital discharge. We believe this to be the case since length of stay in the ICU was not different among the gait speed tertiles in the present study. Indeed, transitioning patients from the ICU to the telemetry unit can happen at a regular time following surgery despite ongoing hemodynamic changes. However, transitioning frail patients out of the hospital cannot be done until extended care facilities are able to care for the patient, requiring a certain level of stability. Hence, the ICU length of stay may not be affected by frailty but the hospital length of stay is increased.

Gait speed had no association with AKI (*p*=0.0584) although the largest incidence of AKI was in the slowest gait speed tertile. The mortality rate in the study cohort was 2.1% (7/333), which correlates to other database rates of cardiac surgical mortality. Notably, the largest percentage of overall deaths (6.5%) was in the slowest gait speed group. However, due to the low number of deaths (*n*=7), this association may be underpowered. As stated previously, the current investigation did find a significant association between mortality and cardiogenic shock (*p*=0.0033), but not vasogenic shock (*p*=0.1340).

### 5.1. Future Directions: Prehabilitation and Discharge Planning

Patients with the slowest gait speed were significantly older. The proportion of elderly patients presenting for cardiac surgery is increasing [3]. While age is not a prerequisite for frailty, certainly frailty is a problematic expression of an aging population. With the proportion of older persons accelerating rapidly worldwide from 461 million people older than 65 years in 2004 to an estimated 2 billion people by 2050, it is important to consider the implications of this population when planning the delivery of healthcare [17, 18].

While age cannot be modified prior to surgery, some preexisting comorbidities that are related to slow gait speed can be optimized if recognized. For example, prehabilitation can improve cardiac surgical outcomes [19, 20]. One recent study found that a home-based exercise program improved frailty scores and walking tests in patients awaiting cardiac surgery [20]. A large meta-analysis demonstrated that prehabilitation programs decreased the number of postcardiac surgical complications [21]. Future studies should aim at solidifying comorbidities that are most often associated with frailty as many may be amenable to attenuation and optimization prior to surgery which would translate into surgical outcome improvements.

## 6. Limitations

The present study has some limitations including a small incidence of adverse events encouraging the repetition of our results with higher powered reproducibility studies to further assess the association of gait speed and adverse cardiac surgical outcomes. It is possible that we did not find an association with gait speed and shock due to the relatively smaller sample size in the slow tertile (*n*=31).

Another limitation is that some of the frailest patients may have been excluded in the analysis due to lack of gait speed assessments prior to surgery. Although considered routine practice to obtain a gait speed study prior to surgery, there are multiple reasons why this did not take place. For example, patients who were unable to ambulate were excluded in the analysis but would likely be considered frail. This would have the potential to modify our results had these patients indeed been frailer than other patients. Along these same lines, some patients did not receive gait speed tests due to lack of a preoperative surgical clinic visit due to direct transfer from an outside hospital for urgent or emergent cardiac surgery. Lack of personnel to administer gait speed studies may have played a role in this low percentage of patients who received this test.

The need for vasopressor support to maintain a mean arterial pressure of 65 mmHg is the commonly utilized definition for vasogenic shock [22]; this single criterion led to inclusion of numerous patients who may have only had vasogenic shock for a short period of time. Notably, 55% of our patients met criteria for vasogenic shock, which we believe to be accurate for a cardiac surgical population, but may not convey the risk associated with prolonged vasogenic shock. For future studies, investigators may wish to define vasogenic shock as a need for vasopressors for a predetermined duration following cardiac surgery. The definition for cardiogenic shock utilized is consistent with the validated STS definition of cardiogenic shock [15].

## 7. Conclusion

While many previous authors have reported adverse outcomes following cardiac surgery associated with gait speed, the exact etiologies of these outcomes have not been investigated. In this exploratory study, we did not find an association of gait speed with cardiogenic and/or vasogenic shock following cardiac surgery. Thus, other factors should be investigated as potential etiologies of adverse outcomes in patients with slow gait speed undergoing cardiac surgery.

To our knowledge, this is the first study to demonstrate a significant association between preoperative gait speed and increased length of hospital stay following cardiac surgery. Future studies should investigate utilization of *prehabilitation* in frail cardiac surgical patients to potentially decrease hospital length of stays.

## Figures and Tables

**Table 1:** Baseline characteristics for the study patients stratified into tertiles of gait speeds.

	Slow (<0.83 m/s) *n*=31	Intermediate (0.83–1 m/s) *n*=216	Fast (>1 m/s) *n*=86	*p* value
*Age (median (Q1, Q3))*	70.00 (65.00, 76.50)	64.00 (55.00, 75.00)	58.00 (50.25, 67.00)	<0.0001
*Gender (%)*				0.1530
Female	12 (38.7)	57 (26.4)	18 (20.9)	
Male	19 (61.3)	159 (73.6)	68 (79.1)	
*STS score (median (Q1, Q3))*	2 (1, 3)	1(1, 3)	1 (0, 2)	0.0019
*Procedure (%)*				0.7724
Aortic	4 (12.9)	30 (13.9)	14 (16.3)	
Isolated CABG	9 (29.0)	86 (39.8)	33 (38.4)	
Isolated valve	6 (19.4)	50 (23.1)	21 (24.4)	
CABG valve	7 (22.6)	30 (13.9)	12 (14.0)	
Others	5 (16.1)	20 (9.3)	6 (7.0)	
*Emergent/urgent (%)*	3 (9.7)	2 (0.9)	3 (3.5)	0.0095
*BMI (mean (SD))*	31.00 (5.24)	29.95 (6.85)	28.42 (4.63)	0.0715
*Race (%)*				0.8274
White	23 (79.3)	175 (85.4)	72 (85.7)	
Black	3 (10.3)	15 (7.3)	5 (6.0)	
Others	3 (10.3)	15 (7.3)	7 (8.3)	
*Diabetes (%)*	12 (38.7)	66 (30.6)	26 (30.2)	0.6398
*CHF (%)*	11 (35.5)	45 (20.8)	28 (32.6)	0.0410
*Preoperative systolic heart failure (%)*	7 (23.3)	63 (30.0)	18 (21.2)	0.2698
*Prior stroke (%)*	3 (9.7)	9 (4.2)	3 (3.5)	0.3078
*Peripheral arterial disease (%)*	2 (6.5)	19 (8.8)	5 (5.8)	0.6546
*Preoperative dysrhythmia (%)*	6 (19.4)	47 (21.8)	10 (11.6)	0.1274
*Chronic lung disease (%)*	21 (67.7)	115 (53.2)	27 (31.4)	0.0003
*Tobacco abuse (%)*	10 (32.3)	101 (46.8)	35 (40.7)	0.2489
CPB minutes (mean (SD))	117.48 (64.86)	105.12 (38.87)	112.88 (44.89)	0.1775

BMI = body mass index; SD = standard deviation; CHF = congestive heart failure; CBP = cardiopulmonary bypass time.

**Table 2:** Outcomes stratified by gait speed tertiles.

	Slow (<0.83 m/s) *n*=31	Intermediate (0.83–1 m/s) *n*=216	Fast (>1 m/s) *n*=86	*p* value^*∗*^
*Primary outcomes*				
Postoperative vasogenic shock (%)	13 (41.9)	128 (59.3)	42 (48.8)	0.0805
Postoperative cardiogenic shock (%)	12 (38.7)	65 (30.1)	22 (25.6)	0.3832

*Secondary outcomes*				
Both cardiogenic and vasogenic shocks	8 (25.8)	51 (23.6)	12 (14.0)	0.1474
30-day mortality	2 (6.5)	3 (1.4)	2 (2.3)	0.1213
ICU hours (median (Q1, Q3))	52.50 (27.65, 89.03)	49.00 (28.50, 73.55)	46.75 (25.60, 52.60)	0.0777
Hospital length of stay (days) (median (Q1, Q3))	7.00 (5.00, 10.00)	6.00 (5.00, 7.00)	5.00 (4.00, 6.00)	0.0050
Surgical wound infection	1 (3.2)	4 (1.9)	0 (0.0)	0.3417
Acute kidney injury	7 (22.6)	23 (10.6)	7 (8.1)	0.0844
Reoperation for cardiac reason	3 (9.7)	11 (5.1)	5 (5.8)	0.5881
Prolonged ventilation	5 (16.1)	15 (6.9)	4 (4.7)	0.1027
Postoperative atrial fibrillation	7 (22.6)	41 (19.0)	16 (18.6)	0.8806
Need for pacemaker	5 (16.1)	8 (3.7)	2 (2.3)	0.0298
Postoperative stroke	0 (0.0)	1 (0.5)	2 (2.3)	0.4010

^*∗*^Significance level *p*=0.025 with two primary outcomes; ICU = intensive care unit; prolonged ventilation is defined as > 24 hours.

**Table 3:** Association of 30-day mortality and postoperative cardiogenic and vasogenic shock.

	30-day mortality		*p* value^*∗*^
	Yes	No	
*Vasogenic shock*			
Yes	6 (3.28%)	177 (96.72%)	0.1340
No	1 (0.67%)	149 (99.33%)	

*Cardiogenic shock*			
Yes	6 (6.06%)	93 (93.94%)	0.0033
No	1 (0.43%)	233 (99.57%)	

^*∗*^Fisher's exact test.

## Data Availability

The data used to support the findings of this study are available from the corresponding author upon request.
